# Effect of Silver Dopants on the ZnO Thin Films Prepared by a Radio Frequency Magnetron Co-Sputtering System

**DOI:** 10.3390/ma10070797

**Published:** 2017-07-14

**Authors:** Fang-Cheng Liu, Jyun-Yong Li, Tai-Hong Chen, Chun-How Chang, Ching-Ting Lee, Wei-Hua Hsiao, Day-Shan Liu

**Affiliations:** 1 Institute of Electro-Optical and Materials Science, National Formosa University, Yunlin 63201, Taiwan; chengliuxd@gmail.com (F.-C.L.); 10476110@gm.nfu.edu.tw (J.-Y.L.); s706802000213@gmail.com (W.-H.H.); 2 Additive Manufacturing and Laser Application, Industrial Technology Research Institute, Tainan 73445, Taiwan; tsaicc1221@gmail.com (T.-H.C.); a0922639175@gmail.com (C.-H.C.); 3 Institute of Microelectronics, National Cheng Kung University, Tainan 70101, Taiwan; tsaicc@ee.ncku.edu.tw

**Keywords:** Ag-ZnO co-sputtered film, radio frequency magnetron co-sputtered system, Ag acceptors, Ag aggregations, *p*-type conduction

## Abstract

Ag-ZnO co-sputtered films at various atomic ratios of Ag (Ag/(Ag + Zn) at.%) were prepared by a radio frequency magnetron cosputtering system, using the co-sputtered targets of Ag and ZnO. The activation of the Ag acceptors (Ag_Zn_) and the formation of the Ag aggregations (Ag^0^) in the ZnO matrix were investigated from XRD, Raman scattering, and XPS measurements. The Ag-ZnO co-sputtered film behaving like a *p*-type conduction was achievable after annealing at 350 °C under air ambient for 1 h.

## 1. Introduction

Zinc oxide (ZnO) is a multi-functional material of the II–VI group for its wide and direct band gap (*E_g_* ~ 3.37 eV) with a large exciton binding energy of 60 meV at room temperature. It also has the advantages of excellent resistance to radiation damage, suitability for the wet-etching process, physical and chemical stability, high oxidative capacity, low cost, and availability. In recent decades, ZnO has become a very encouraging material utilized in multiple fields, such as solar cells, transparent conductive contacts, light emitting devices, spintronic devices, laser deflectors, paints, antibacterial agents, bio-sensors, piezoelectric transducers, and gas sensors [1,2,3,4,5]. Among these devices and component applications, many efforts have been made to modify the optical and/or electrical properties of ZnO through doping, ion irradiation, etc., so that it can be used more widely in research fields undoped, as untreated ZnO is generally inactive to carrier transmission and ineffective for solar energy adsorption. For instance, when the ZnO material is applied as a photocatalyst to promote the decomposition of the organic pollutants, less than 5% of the solar spectrum at the Earth’s surface consisting of the UV wavelengths can drive the photocatalytic process because the absorption edge is constrained by the natural band gap of ZnO. Band gap modification of ZnO through metal doping is one of the promising approaches to extend the absorption of light into the visible wavelengths. In addition, these metal dopants have also demonstrated properties of electron sinks to increase the life-span of the photo-generated electron-hole pairs [6,7,8]. On the other hand, when ZnO is applied to optoelectronic device fabrication, one of the major obstacles for realizing ZnO-based optoelectronic devices is the difficulty in achieving quality *p*-type ZnO because of the self-compensation effect that originates from native defects, as well as the limited solubility and inactivation of the acceptor dopants in the ZnO material. Although steady progress in doping ZnO with *p*-type, using group-I elements for zinc sites and/or group-V elements for oxygen sites, has been reported [9,10,11], the reproducibility of the *p*-type ZnO still is challenging due to the group-I and group-V dopants being prone to forming the interstitial site or antisite defects, respectively, due to the significant size-mismatch to the lattice atoms. Recently, group-IB elements (such as Ag and Cu) with less size-mismatch and larger ionization energy than the group-I elements were announced as an alternative dopants to achieve a quality *p*-type ZnO [12,13,14]. In addition to realize *p*-type ZnO, nanoparticles- or nanorods-ZnO prepared using group-IB elements have also been applied to enhance the performance of the resulting ZnO-based optoelectronic devices via the localized surface plasmon [15,16,17,18]. Among these group-IB elements, silver has excellent electrical, optical, and chemical properties for promoting the photocatalytic activity, conductive type, and luminescence emission of the ZnO material. Accordingly, insights into the activation of the Ag dopants in the ZnO matrix are critical.

Silver-doped ZnO (referred to as Ag-ZnO, hereafter) have been synthesized using several technologies, such as photochemical, solvothermal, and pulse laser deposition [19,20,21]. It is also acceptable to prepare using sputtering technology, which is widely used in the coating industry [22,23]. In this work, we used a radio frequency (RF) magnetron co-sputtering system, which has the advantage of simple and in situ control on the elemental composition of the resulting film over the conventional sputtering system, to prepare Ag-ZnO co-sputtered films at various Ag atomic ratios. Electrical, optical, and material properties of the Ag-ZnO co-sputtered films at various theoretical Ag atomic ratios were measured to understand the behavior of the Ag dopants in the ZnO matrix. The origin responsible for the change in the conduction type of the Ag-ZnO co-sputtered films can be reasonably explained by the evolutions in their crystalline structures and chemical bond configurations.

## 2. Experimental Procedure

**Figure 1:**
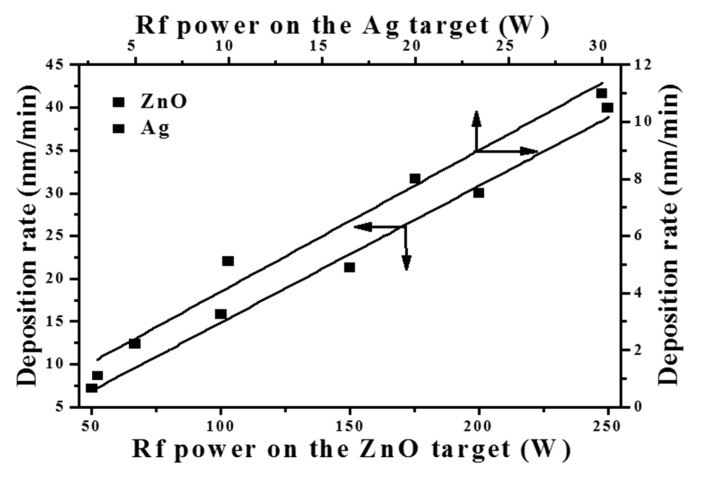
Deposition rates of the single ZnO and Ag films as functions of the RF power supplied on the ZnO and Ag targets, respectively.

The RF magnetron co-sputtering system was constructed from a dual RF power supply that generated two different RF powers with synchronized phases. The configuration of the RF magnetron co-sputtering chamber has been illustrated elsewhere [24]. High-purity ZnO (99.99%) and metallic Ag (99.99%) were selected as the co-sputtering targets. Figure 1 depicts the deposition rates of the single ZnO and metallic Ag films as functions of the RF power supplied to the ZnO and Ag targets, respectively. To deposit Ag-ZnO films at various Ag doping levels, the RF power supplied to the ZnO target was fixed at 250 W while that supplied to the Ag target was varied from 2 to 6.8 W. The theoretical Ag atomic ratios [Ag/(Ag + Zn) at.%] introduced into the ZnO films could be evaluated from the following expression similar to our previous reports [24,25,26]:

(1) $$\frac{D_{1}\times A\times d_{1}}{M_{1}}:\frac{D_{2}\times A\times d_{2}}{M_{2}}=P:Q,$$

where *D*_1_ and *D*_2_ (nm/min), respectively, are the deposition rates of the single Ag and ZnO films prepared at specific RF powers; *A* (nm^2^) is defined as the cross-section area of the substrate surface; *d*_1_ and *d*_2_ (g/cm^3^) are related to the density of the Ag (10.49 g/cm^3^) and ZnO (5.66 g/cm^3^) materials; *M*_1_ and *M*_2_ (g/mole) are the atom and molecular weights of the Ag and ZnO materials; *P* and *Q* (mole) are the mole ratios of Ag and Zn atoms in the co-sputtered films. According to the deposition rates of the single ZnO and Ag films prepared at each RF power, shown in Figure 1, we controlled the Ag-ZnO co-sputtered films at the theoretical Ag atomic ratios of 1, 3, 5, and 8 at.%. The films’ thickness was fixed at about 200 nm. All the films were grown onto *n*-type Si (100) substrates at room temperature. Moreover, in order to measure the films’ optical transmittance at visible and ultraviolet wavelengths, one set of the films were deposited onto the glass substrates. To activate the Ag dopants and facilitate the crystalline re-growth, all the undoped ZnO and Ag-ZnO co-sputtered film were post-annealed at 350 °C for 1 h under ambient air.

Film thickness of these films was measured using a surface profile system (Dektak 6M, Veeco, New York, NY, USA). Resistivity, carrier concentration, and hall mobility were measured using the van der Pauw method with a Hall measurement system (HMS-5000, Ecopia, Anyang, Korea). Optical transmittance was measured by a UV-VIS spectrophotometer (UVD-3500, Labomed, Inc., Los Angeles, CA, USA). The surface morphologies were examined using a field emission scanning electron microscopy (FE-SEM; JSM-6700F, JEOL, Tokyo, Japan) with the accessory of the energy-dispersive X-ray spectroscopy (EDS). Evidence of the dopants activation in the ZnO film resulted in the evolutions on the material properties were conducted from X-ray diffraction (XRD; D-500, Siemens, Munich, Germany) patterns, Raman spectra (MRI-A003, ProTrusTech, Tainan, Taiwan), and X-ray photoelectron spectroscopy (XPS; Quantera SXM™, ULVAC-PHI, Kanagawa, Japan).

## 3. Results and Discussion

**Figure 2:**
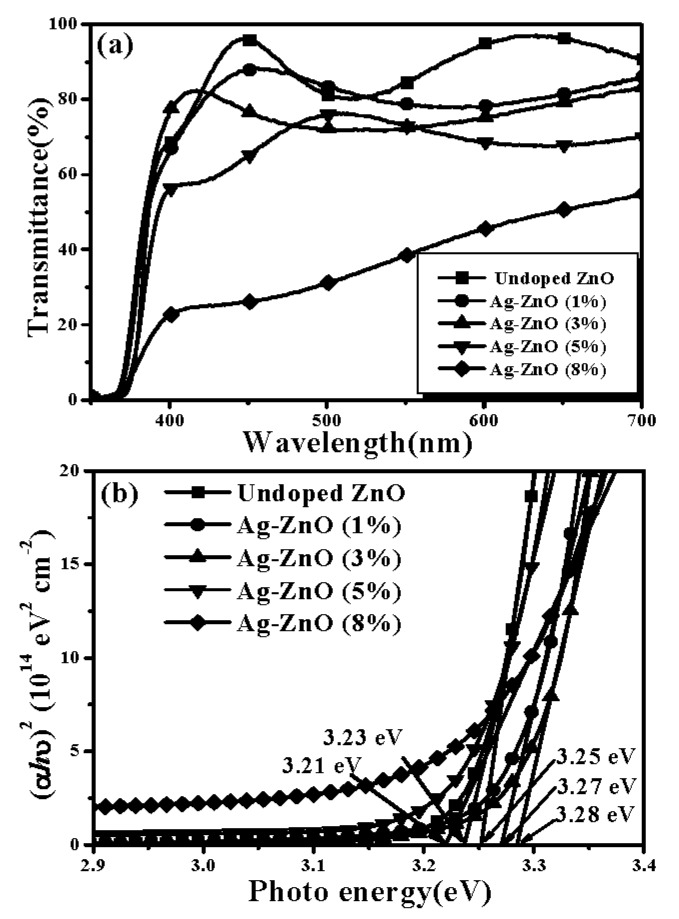
(**a**) Optical transmittance spectra and (**b**) the plot of (*αhν*)^2^ versus the photon energy of the undoped ZnO and Ag-ZnO co-sputtered films annealed at 350 °C for 1 h under ambient air.

Table 1 summarizes the electrical properties of the undoped ZnO and ZnO films doped at various theoretical Ag atomic ratios after annealing at 350 °C for 1 h under ambient air, measured using the van der Pauw method at room temperature. The resistivity of the undoped ZnO film was too high to be measured, while these Ag-ZnO films behaved in a different conductive manner after the post-annealing treatment. A *p*-type conductor with a hole concentration of 5.2 × 10^16^ cm^−3^ was obtained from the ZnO film co-sputtered with a theoretical Ag concentration of 1 at.%. The hole carriers were further increased to 5.9 × 10^17^ cm^−3^ as the Ag dopants in the ZnO film reached a theoretical atomic ratio of 3 at.%. However, the conductive type converted into *n*-type with very high electron carriers of 1.9 × 10^20^ cm^−3^ as the ZnO film co-sputtered at a theoretical Ag atom ratio of 5 at.%. In addition, more electron carriers as high as 1.7 × 10^21^ cm^−3^ were measured from the ZnO film doped at a theoretical Ag atom ratio of 8 at.%. The associated optical transmittance spectra are shown in Figure 2a. The undoped ZnO film annealed at 350 °C for 1 h under ambient air had a high average transmittance of about 89% at visible wavelengths (400–700 nm). For the ZnO film co-sputtered with the Ag atoms, the average transmittance at visible wavelengths decreased as more Ag atoms were introduced into the ZnO films, as listed in Table 1. Eventually, the annealed Ag-ZnO film at a theoretical atomic ratio of 8% became semi-opaque with a low average transmittance of about 38%. Figure 2b shows the corresponding optical energy band gap determined from the plot of (*αhν*)^2^ versus the photon energy. Compared to the undoped ZnO film, the onset of the absorption edge in the ultraviolet wavelengths for these annealed Ag-ZnO co-sputtered films initially shifted toward the short wavelength, resulting in a widened optical energy band gap from 3.25 to 3.28 eV as the theoretical Ag dopants reached 3%. Then, a slight redshift on the absorption edge with the optical energy band gap narrowing was observed from the annealed Ag-ZnO co-sputtered film at the theoretical doping level of 5% and 8% (the corresponding optical energy band gaps were 3.23 and 3.21 eV, respectively).

**Figure 3:**
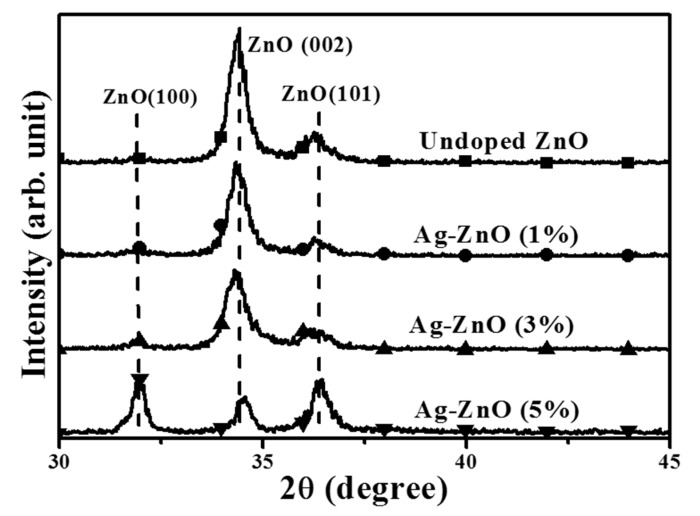
XRD patterns of the undoped ZnO and the co-sputtered Ag-ZnO films at the theoretical Ag atomic ratios of 1%, 3%, and 5%, respectively, after annealing at 350 °C for 1 h under ambient air.

**Figure 4:**
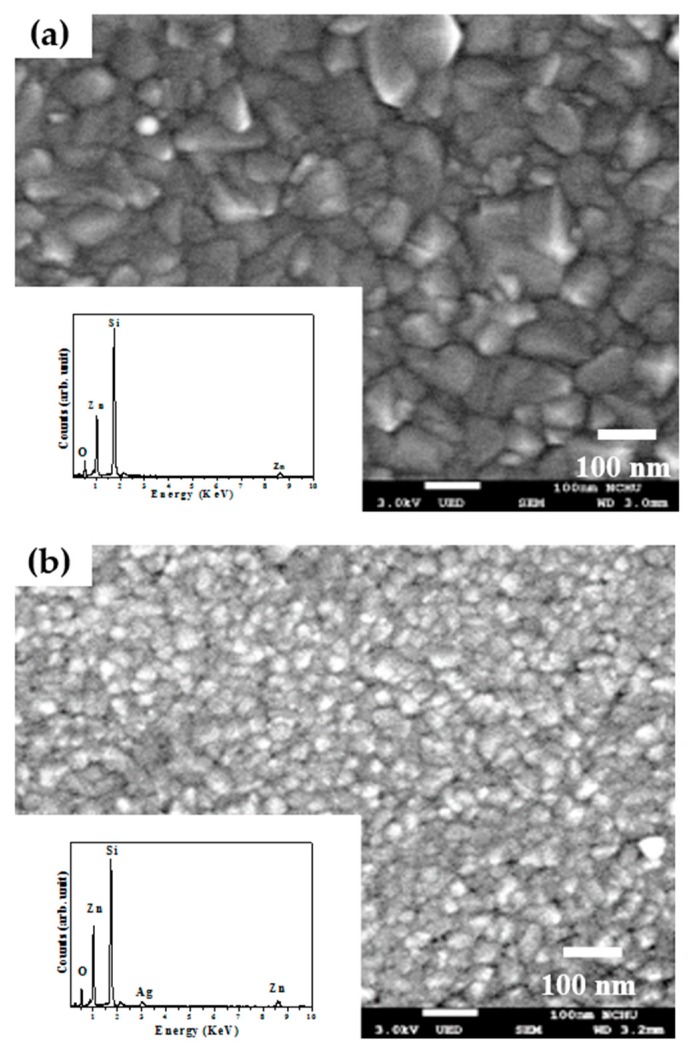
Surface morphologies of the (**a**) undoped ZnO film and (**b**) Ag-ZnO co-sputtered film at a theoretical atomic ratio of 3% after annealing at 350 °C for 1 h under ambient air (the inset figures shows the elemental compositions conducted from EDS measurement).

**Figure 5:**
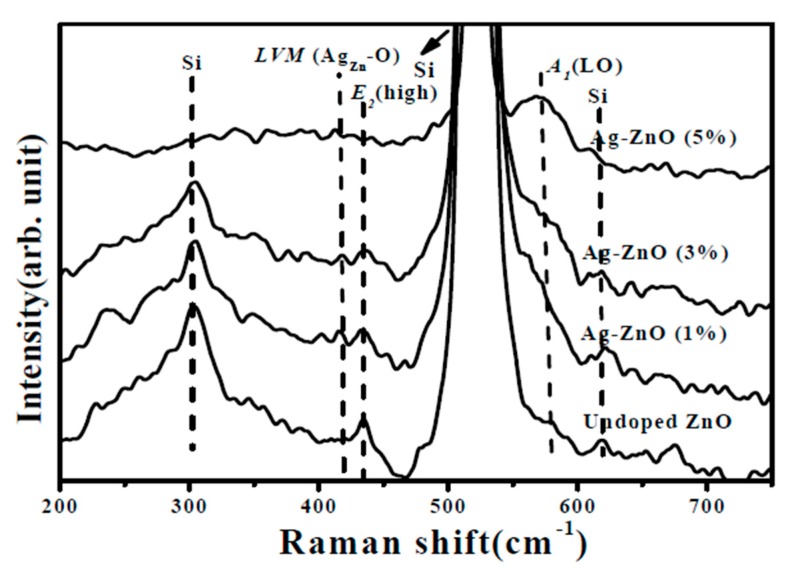
Raman spectra of the undoped ZnO and Ag-ZnO co-sputtered films annealed at 350 °C for 1 h under ambient air.

The crystalline structures conducted from XRD measurements for the undoped ZnO and the Ag-ZnO co-sputtered films at the theoretical Ag atomic ratios of 1%, 3%, and 5%, respectively, after annealing at 350 °C for 1 h under air ambient are shown in Figure 3. All samples behaved like polycrystalline structures identified as the ZnO hexagonal wurtzite type, and no signal related to Ag or its oxides phase was detected. The undoped ZnO film exhibiting the preferred growth orientation along the *c*-axis as evidence of the peak at about 34.38° assigned as ZnO (002) phase according to JCPDS database (JCPDS card no. 36-145) was predominant throughout the XRD pattern. The ZnO (002) phase was also the dominant growth structure as Ag atoms was incorporated into the ZnO films at the theoretical atomic ratios of 1% and 3%. By contrast, two peaks assigned as ZnO (100) and ZnO (101) phase became the dominant signal in the XRD pattern measured from the ZnO film co-sputtered at a theoretical Ag atomic ratio of 5%, revealing the disappearance of the *c*-axis preferred growth orientation. Moreover, compared to the undoped ZnO film, the full width at half maximum (FWHM) of the ZnO (002) peak increased as the Ag atoms doped into the ZnO film increased and slightly shifted on the peak position. Table 2 summarizes the peak position and the FWHM of the ZnO (002) phase as well as the corresponding crystalline size, *D*, evaluated from the FWHM of the preferred orientation ZnO (002) according to the following Debye-Scherer formula:

(2) $$D=\frac{k\lambda }{\beta \cos\theta },$$

where *k* is a constant (*k* = 0.9), *λ* is the wavelength of the X-ray radiation, *β* is the FWHM in radians, and *θ* is the Bragg diffraction angle. The crystalline size growing along the *c*-axis was found to be suppressed as the Ag atoms doped into the ZnO film. The crystalline size apparently decreased from about 19.1 nm to 14.9 nm as the ZnO film co-sputtered at a theoretical Ag atomic ratio of 5% due to the degradation on the *c*-axis growth orientation. In terms of the peak position of the ZnO (002) phase, a theoretical Ag atom ratio of 1% introduced into the ZnO matrix resulted in the peak shifting toward a low 2*θ* value of 34.34°. The reason responsible for the shift of the ZnO (002) peak was likely the activated Ag^1+^ ions substituted for the Zn^2+^ lattice sites (Ag_Zn_) since the ionic radius of the Ag atom (0.126 nm) was higher than that of the Zn atom (0.074 nm). In addition, the activation of the Ag dopants also led to the Ag-ZnO film behaving as a *p*-type conduction. As the theoretical Ag doping level in the ZnO films reached 3% which had a higher hole carriers, the increase in the amounts of the activated Ag acceptors caused a further shift of the ZnO (002) peak toward a low 2*θ* value of 34.32°. In contrast, a high diffraction angle of the ZnO (002) phase at about 34.56° was measured from the ZnO film doped with the Ag atoms at a theoretical atomic ratio of 5% while exhibiting *n*-type degenerated conduction without *c*-axis growth orientation. This indicated that the activation of the Ag acceptor was saturated and another mechanism would be induced as the theoretical Ag dopants reached 5%. Figure 4a,b show the surface morphologies of the undoped ZnO film and the Ag-ZnO co-sputtered film at a theoretical atomic ratio of 3% after annealing at 350 °C for 1 h under air ambient (the elemental compositions conducted from EDS measurement are also shown in the inset figures). Textures with wedge-like grains were observed from the surface of the annealed undoped ZnO film and only the elements of Zn, O, and Si (the signal emerging from the substrate) were measured. By contrast, the grain size distributed over the surface of the annealed Ag-ZnO co-sputtered film was reduced with ambiguous grain boundaries and a weak peak denoted as Ag could be found from the corresponding EDS spectrum. The shrink in the grain size and the disappearance of the surface textures as the silver atoms incorporated into the ZnO film also supported the degradation in the crystalline structure as investigated from the XRD measurements. The vibration properties of the annealed undoped ZnO and Ag-ZnO co-sputtered films investigated using micro-Raman spectroscopy are plotted in Figure 5. The peaks at about 303, 521, and 618 cm^−1^, respectively, were due to scattering from the silicon substrate. The Raman spectrum is an essential and versatile diagnostic study on the crystallization, structural disorder, and defects in micro- and/or nano-structures. Complying with the Raman selection rules in wurtzite crystal structures, two specific lines corresponding to the *E*_2_ high frequency branch and *A*_1_ longitudinal optical modes (denoted as *E*_2_(high) and *A*_1_(LO) in the spectrum) at around 435 and 580 cm^−1^, respectively, were observed in the Raman spectrum of the undoped ZnO sample [27,28,29].

When the ZnO film was doped with the Ag atoms, the *E*_2_(high) mode was gradually decreased and broadened as the theoretical Ag dopants increased, and then this mode was hardly observed in the Raman spectrum of the Ag-ZnO film at an atomic ratio of 5%. As quoted from the previous reports, the *E*_2_(high) phonon mode is related to the crystalline nature, phase orientation, and strain present in the ZnO matrix [30,31,32]. The decrease in the intensity of the *E*_2_(high) signal when the Ag atoms were introduced into the ZnO system was attributed to the disordered crystalline structure of the ZnO film, as confirmed from the degradation of the *c*-axis growth orientation discussed in the XRD patterns. Furthermore, the Ag-ZnO film at an atomic ratio of 5% grew without *c*-axis orientation, resulting in the *E*_2_(high) mode being almost absent in the associated Raman spectrum. In contrast to the change of the *E*_2_(high) phonon, the signal of the *A*_1_(LO) mode became more significant as the Ag atoms doped into the ZnO film increased, and an apparent and wide peak was identified from the Raman spectrum of the ZnO film doped at the Ag level of 5%. The enhancement on the *A*_1_(LO) signal implied the increase of the defects in the ZnO film since the *A*_1_(LO) mode is represented to the defect complexes, such as zinc interstitial (Zn_I_) and oxygen vacancy (V_O_) in the ZnO lattice [33,34]. In addition, another weak peak at around 414 cm^−1^ induced by the localized vibration mode (*LVM*) in the ZnO film appeared in the Raman spectra of the Ag-ZnO co-sputtered film. As referred from the reports [35,36], this *LVM* mode was an indication of the dopant incorporation associated with the Ag ion in substitution for the Zn lattice site in the Zn-O bond configuration (denoted as *LVM*(Ag_Zn_-O)).

**Figure 6:**
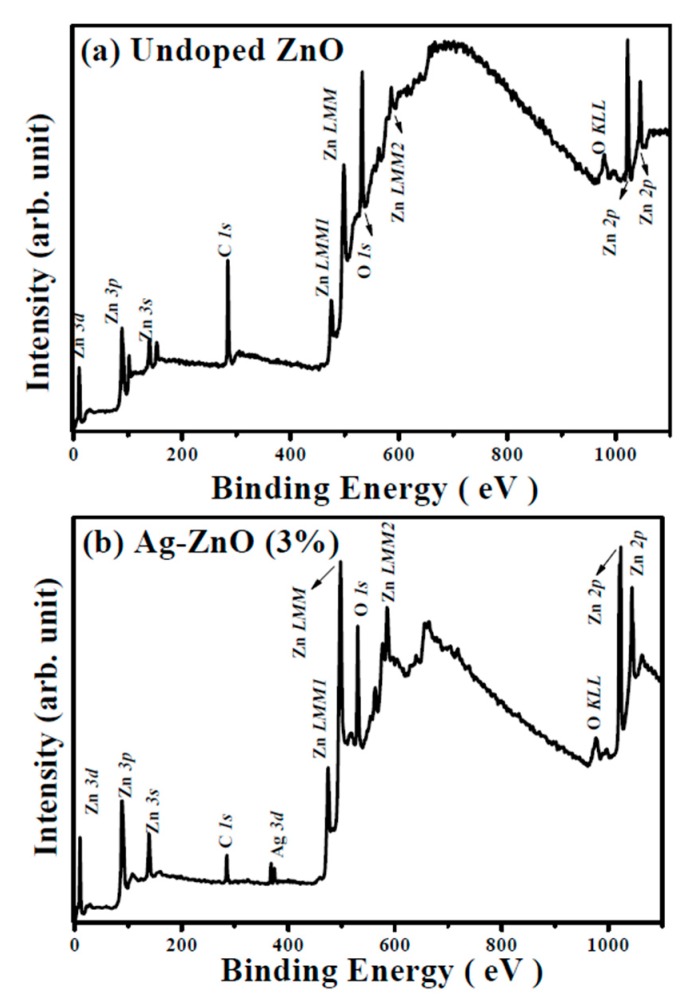
XPS survey spectra taken on the surface of the (**a**) undoped ZnO film and (**b**) Ag-ZnO co-sputtered film at a theoretical atomic ratio of 3% after annealing at 350 °C for 1 h under ambient air.

**Figure 7:**
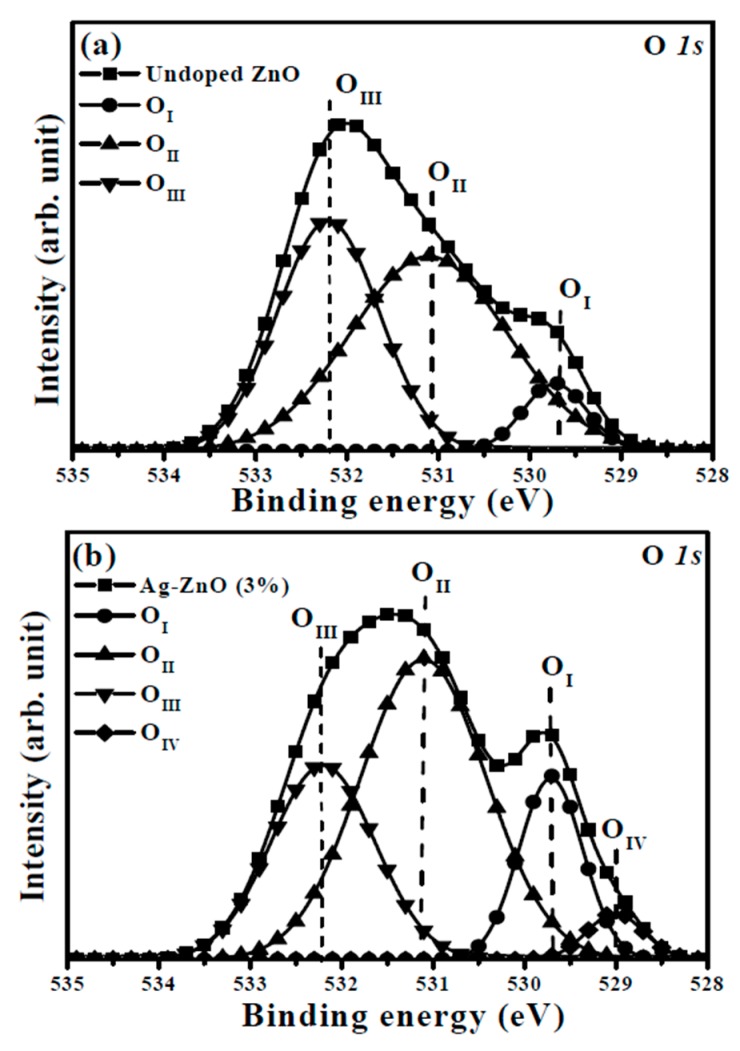
Core level of the O *1s* spectra for the (**a**) undoped ZnO film and (**b**) Ag-ZnO co-sputtered film at a theoretical atomic ratio of 3% after annealing at 350 °C for 1 h under ambient air.

**Figure 8:**
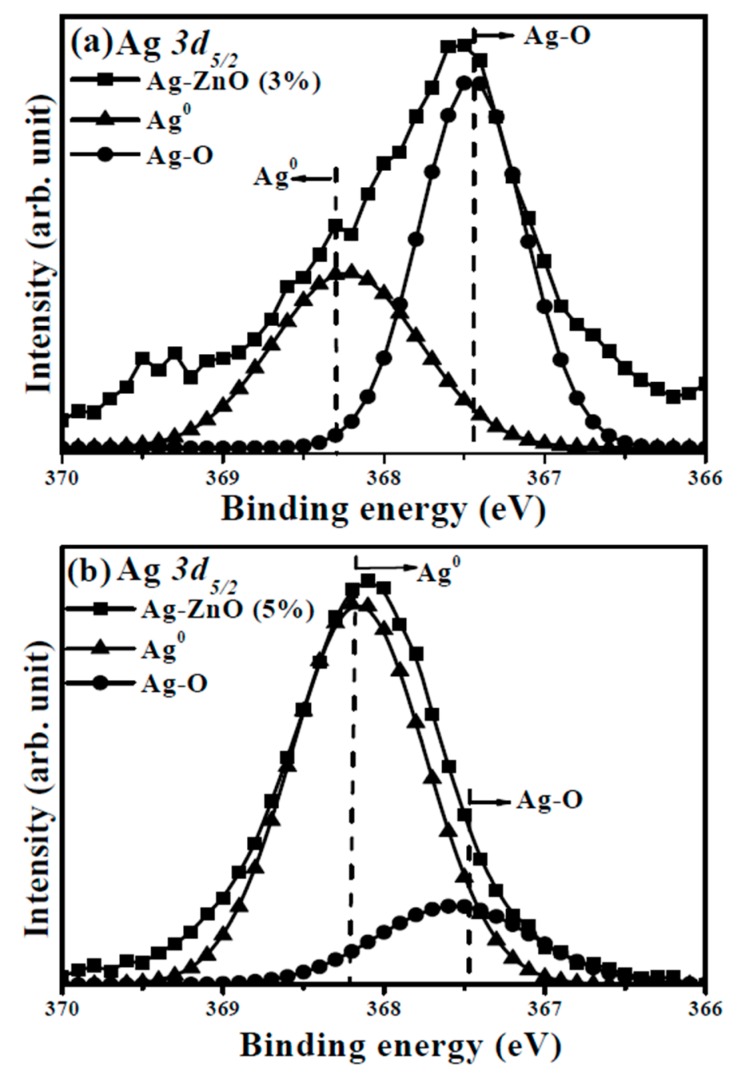
Core level of the Ag *3d*_5/2_ spectra for the Ag-ZnO co-sputtered films at the theoretical atomic ratios of (**a**) 3% and (**b**) 5%, respectively, after annealing at 350 °C for 1 h under ambient air.

Although the activation of the Ag acceptors in the Ag-ZnO co-sputtered films were confirmed by the appearance of the *LVM* signal, only the co-sputtered films at the theoretical Ag atomic ratio of 1% and 3% behaved like a *p*-type conduction. Thus, the chemical bond configurations conducted from the XPS measurement were carried out to understand the mechanism responsible for the conversion from *p*- to *n*-type conduction of the Ag-ZnO co-sputtered film at the theoretical Ag dopants of an atomic ratio of 5%. The XPS survey spectra taken on the surface of the undoped ZnO and Ag-ZnO co-sputtered film at a theoretical atomic ratio of 3% annealed at 350 °C for 1 h under ambient air are shown in Figure 6a,b, respectively. Both spectra were characterized as the peaks of Zn and O elements with the appearance of the C *1s* peak at 284.5 eV for reference. Two peaks at about 368 and 374 eV assigned as the signal related to Ag *3d*_5/2_ and Ag *3d*_3/2_ were observed only in the spectrum of the Ag-ZnO co-sputtered film. Figure 7a,b show the high resolution of the O *1s* spectra for further realizing the evolution of the oxidized states when Ag was incorporated into the ZnO matrix. As can be seen in Figure 7a, the core level of the O *1s* for the undoped ZnO film exhibited a peak at 532.0 eV with asymmetric behavior, which could be deconvoluted into three types of oxygen groups. The peaks at around 529.7 eV and 531.1 eV (denoted as O_I_ and O_II_ in the figure) were respectively attributed to the oxygen ions in the fully oxidized surrounding (i.e., Zn-O bonding) and in oxygen-deficient regions (i.e., oxygen vacancy), whereas the peak at about 532.2 eV (denoted as O_III_) was related to the hydroxyl (OH) group or the loosely bound oxygen on the surface [21,34,37,38]. Compared to the undoped ZnO film, the O *1s* peak measured from the surface of the ZnO film co-sputtered at the Ag atomic ratio of 3% shifted to about 531.5 eV with a significant satellite peak at 529.7 eV and a tail extending to low binding energy. This curve could be deconvoluted into the above-mentioned three oxidized states with an additional weak peak at 528.9 eV (denoted as O_IV_). As indicated in the previous reports [39,40,41], this oxidized state observed only in the Ag-ZnO co-sputtered film was the contribution of the atomic oxygen with an ionic Ag-O bond, which implied the activated Ag dopants (Ag_Zn_) as a substitution for the lattice Zn in the ZnO matrix. Additionally, in agreement with [21], the incorporation of the Ag atoms in the ZnO film also led to the reduction in the oxygen vacancy-related defects as evidence of the decrease in the ratio of the peak area (O_II_/(O_I_ + O_II_ + O_IV_)). Although the formation of the Ag-O chemical bond and the suppression on the native oxygen vacancy donors confirmed from the investigation of the O *1s* core level were both helpful for realizing a *p*-type Ag-ZnO co-sputtered film, an *n*-type Ag-ZnO still was measured when the theoretical Ag dopants reached 5%. The core level of the Ag *3d*_5/2_ for the Ag-ZnO co-sputtered films at the atomic ratios of 3% and 5%, shown in Figure 8a,b, respectively, are given to elucidate the conversion of the conduction type. The peak of the Ag *3d*_5/2_ shifted from 367.5 eV for the Ag-ZnO (3%) co-sputtered film to 368.1 eV for the Ag-ZnO (5%) co-sputtered film. Such an asymmetric peak could be deconvoluted into two peaks at about 367.4 and 368.2 eV, which were, in turn, ascribed to the bonds associated with the metallic and ionic Ag (denoted as Ag^0^ and Ag-O in the figure), respectively [21,40,42,43]. Clearly, the Al-ZnO (3%) co-sputtered film mainly contained the Ag-O chemical bond, whereas the metallic Ag-Ag bond dominated over the Ag-ZnO (5%) co-sputtered film. Combined with the electrical property, the achievement of the *p*-type conduction for the Ag-ZnO (3%) co-sputtered film was attributed to the efficient activation of the Ag acceptors (Ag_Zn_) as evidence of most of the Ag dopants forming the Ag–O chemical bonds. However, as the theoretical Ag doping level reached 5%, the overwhelming metallic Ag bond that was closely linked to the aggregation of the Ag dopants led the film to perform *n*-type degenerated conduction. These Ag aggregations would also constrict the growth of the ZnO matrix, thereby resulting in the decrease of the *c*-axis lattice constant and a significant degradation of the crystalline structure, as shown in Figure 3. In addition, the Ag-ZnO co-sputtered films at theoretical atomic ratios of 3% and 5 at.% were about 0.3 and 1.1 at.%, respectively, as determined by the XPS measurements. The significant discrepancy between the actual and theoretical values in the Ag-ZnO co-sputtered films could be attributed to the poison of the Ag target during the co-sputtering deposition.

## 4. Conclusions

Various Ag atoms doped into the ZnO films were prepared by an RF magnetron co-sputtering system, using Ag and ZnO targets. The conduction type of the Ag-ZnO co-sputtered film was controlled by altering the Ag dopants in the ZnO film with an additional post-annealed treatment at 350 °C for 1 h under air ambient. For the Ag-ZnO co-sputtered films at atomic ratios of 1% and 3%, *p*-type conduction was linked to the formation of the Ag–O chemical bond originating from the activation of Ag acceptors substituted for the Zn^2+^ lattice sites (Ag_Zn_) through the XPD, Raman scattering, and XPS investigations. However, as the Ag atoms introduced into the ZnO film reached a theoretical atomic ratio of 5%, the conduction type converted into the generated *n*-type conduction. The mechanism responsible for the conduction conversion was the large amounts of the metallic Ag bond (Ag^0^) appearing on the ZnO matrix, which was correlated to the formation of the Ag aggregations due to the excess incorporation of the Ag atoms. The control maintained over the conduction type of the Ag-ZnO film prepared using RF magnetron co-sputtering technology was very promising for realizing a ZnO-based homojunction optoelectronic device. In addition, the aggregation of the doping Ag in the ZnO matrix might be advantageous for preventing the recombination of the photogenerated electron-hole pairs in photocatalytic applications.

## Figures and Tables

**Table 1:** Electrical properties of the undoped ZnO and ZnO films doped at various theoretical Ag atomic ratios annealed at 350 °C for 1 h under ambient air.

Sample	*n* or *p* (cm^−3^)	*μ* (cm^2^/V s)	*ρ* (Ω cm)	*T*_avg_ (%)
Undoped ZnO	N/A	N/A	N/A	89
Ag-ZnO (1%)	5.2 × 10^16^	3.3	24.1	81
Ag-ZnO (3%)	5.9 × 10^17^	2.9	3.5	76
Ag-ZnO (5%)	−1.9 × 10^20^	1.9	1.8 × 10^−2^	69
Ag-ZnO (8%)	−1.7 × 10^21^	1.5	2.4 × 10^−3^	38

**Table 2:** Peak position and the FWHM of the ZnO (002) phase, as well as the corresponding crystalline size, *D*, for the undoped ZnO and the Ag-ZnO co-sputtered films annealed at 350 °C for 1 h under ambient air.

Sample	2*θ* (deg.)	FWHM (deg.)	*D* (nm)
Undoped ZnO	34.48	0.43	19.1
Ag-ZnO (1%)	34.34	0.48	17.2
Ag-ZnO (3%)	34.32	0.50	16.6
Ag-ZnO (5%)	34.56	0.56	14.9
